# Lung Involvement in Adult T-Cell Lymphoma Diagnosed Using Bronchoscopic Cryobiopsy: A Case Report and Review of the Literature

**DOI:** 10.3390/medicina59112015

**Published:** 2023-11-16

**Authors:** Yasuhiro Tanaka, Takashi Kido, Noriho Sakamoto, Atsuko Hara, Takeharu Kato, Ritsuko Miyashita, Mutsumi Ozasa, Takatomo Tokito, Daisuke Okuno, Kazuaki Takeda, Hirokazu Yura, Shinnosuke Takemoto, Takahiro Takazono, Hiroshi Ishimoto, Yasushi Obase, Yuji Ishimatsu, Yasushi Miyazaki, Hiroshi Mukae

**Affiliations:** 1 Department of Respiratory Medicine, Nagasaki University Hospital, Nagasaki 852-8102, Japan; yasuhiro.t.9462@gmail.com (Y.T.); nsakamot@nagasaki-u.ac.jp (N.S.); clover0409ah@gmail.com (A.H.); r.miya@nagasaki-u.ac.jp (R.M.); ozasamu@nagasaki-u.ac.jp (M.O.); tomo.kyudo@gmail.com (T.T.); vkvkv10101@gmail.com (D.O.); k-takeda@nagasaki-u.ac.jp (K.T.); h-yura@nagasaki-u.ac.jp (H.Y.); shinnosuke-takemoto@nagasaki-u.ac.jp (S.T.); takahiro-takazono@nagasaki-u.ac.jp (T.T.); h-ishimoto@nagasaki-u.ac.jp (H.I.); obaseya@nagasaki-u.ac.jp (Y.O.); hmukae@nagasaki-u.ac.jp (H.M.); 2 Department of Hematology, Nagasaki University Hospital, Nagasaki 852-8102, Japan; tkatou@nagasaki-u.ac.jp (T.K.);; 3 Department of Infectious Diseases, Nagasaki University Graduate School of Biomedical Sciences, Nagasaki 852-8102, Japan; 4 Department of Nursing, Nagasaki University Graduate School of Biomedical Sciences, Nagasaki 852-8102, Japan; yuji-i@nagasaki-u.ac.jp

**Keywords:** adult T-cell lymphoma, pulmonary lymphoma, transbronchial lung cryobiopsy

## Abstract

The diagnosis of pulmonary lymphoma using small tissue samples is difficult and often requires surgical procedures; thus, a less invasive sampling method is desirable. Moreover, pulmonary involvement in adult T-cell lymphoma (ATL) is often difficult to diagnose, especially in cases without characteristic flower cells. Here, we present the case of a 78-year-old man, in whom pathological examination of the transbronchial lung biopsy (TBLB) specimen did not reveal malignant findings; therefore, transbronchial lung cryobiopsy (TBLC) in combination with endobronchial ultrasonography (EBUS) was used to diagnose ATL based on the pathological findings. A literature review identified 18 cases of pulmonary lymphomas diagnosed using TBLC. Among the 19 cases, including our own, 16 cases were of B-cell lymphoma (84.2%), and the present case is the first case of ATL diagnosed using TBLC. Eighty percent of the cases underwent a biopsy (more than two samples) of the middle or lower lobe and were diagnosed without major complications. EBUS was used with TBLC in three cases to identify the location of the pulmonary lesions. In the present case, EBUS was also useful for avoiding vascular biopsy. Although large-scale prospective studies are required to establish precise guidelines for diagnosing pulmonary lymphomas using TBLC, our case report and review contributes to a deeper understanding of the diagnosis of rare diseases.

## 1. Introduction

Adult T-cell lymphoma (ATL) is a peripheral blood T-cell hematologic tumor caused by human T-cell leukemia virus type 1 (HTLV-1). Approximately 3–5% of HTLV-1 carriers develop T-cell tumors with a poor prognosis [1]. ATL is a globally rare T-cell lymphoma, although it is prevalent in certain areas, including western Japan [2]. In a survey of lymphoma cases in Japan occurring over a 2-year period (2006 and 2007), ATL accounted for 910 cases (11.3%) [3]. Given the poor prognosis for aggressive ATL, with a median survival rate of 11 months and a 5-year survival rate of 14% [4], early diagnosis and appropriate chemotherapy are essential.

If there is minimal lymphocyte atypia, pathologically diagnosing pulmonary lymphoma or lymphoma lung infiltration becomes challenging. Methods such as transbronchial lung biopsy (TBLB) or computed tomography (CT)-guided lung biopsy becomes challenging due to the small size of the tissue samples, comprising a mixture of infiltrates, including neoplastic lymphoid cells alongside other inflammatory cells. For the diagnosis of pulmonary lymphomas, a diagnostic surgical lung biopsy (SLB) is necessary in 55.7–100% of patients [5]. Therefore, a less-invasive diagnostic procedure is desirable. Transbronchial lung cryobiopsy (TBLC) has attracted considerable attention in recent years due to its ability to obtain large samples and its usefulness for diagnosing diffuse lung diseases that are difficult to diagnose using conventional forceps-biopsy samples [6]. In addition, TBLC is safer and results in fewer complications and mortality than SLB; thus, early treatment can be achieved [7]. However, the utility and safety of TBLC for lymphoma diagnosis requires further investigation. Here, we present a case of ATL diagnosed using TBLC, which, to the best of our knowledge, is the first such case to be reported. The report includes a review of 18 cases of pulmonary lymphomas diagnosed using TBLC and will contribute to a deeper understanding of rare disease diagnoses.

## 2. Case Report

**Figure 1:**
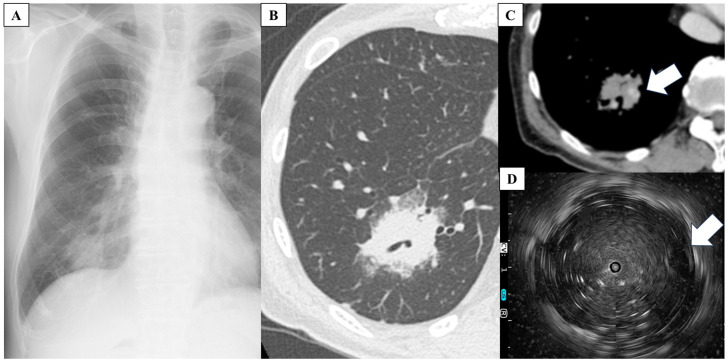
(**A**) Chest radiograph shows a mass in the right lower lung field. (**B**,**C**) High-resolution computed tomography of the chest shows a 30 mm mass with a cavity in the posterolateral lower lobe of the right lung with a bronchial artery (white arrow) inside. (**D**) Endobronchial ultrasonography shows the bronchial artery (white arrow).

A 78-year-old Japanese man visited the hospital with a 2-month history of nonproductive cough and general malaise. He had a 62.5 pack-year smoking history until the age of 50 years and cough-variant asthma. Chest radiography and high-resolution CT (HRCT) showed consolidation in the right lower lung lobe. Antibiotic therapy was initiated after sputum collection; however, no causative microorganisms were identified on culture, and radiological findings worsened. Although bronchoscopy was performed, no malignant findings were observed in the lung-tissue samples obtained using TBLB, and a definitive diagnosis could not be made. The patient was referred to our hospital for further evaluation. His vital signs were as follows: temperature, 36.7 °C; heart rate, 87 beats/min; respiratory rate, 16 breaths/min; blood pressure, 134/86 mmHg; and oxygen saturation, 98% on room air. Chest auscultation revealed fine crackles on the dorsal surface of the right lung. Laboratory tests showed normal results for routine blood work, including a white blood cell count of 4200/μL (lymphocytes, 19.9%; neutrophils, 65.6%; monocytes, 12.4%; eosinophils, 1.4%), a hemoglobin concentration of 12.9 g/dL, and a platelet count of 14.4 × 10^4^/μL. The anti-HTLV-1 antibody level was elevated at 60.15 s/co (reference value, <1.00 s/co). Additionally, the soluble interleukin 2 receptor level was elevated at 3278 U/mL (reference range, 121–613 U/mL). However, the C-reactive protein (0.11 mg/dL), pro-gastrin-releasing peptide (78.2 pg/mL), squamous cell carcinoma antigen (1.0 ng/mL), and carcinoembryonic antigen levels (<1.7 ng/mL) were within the respective normal ranges. Furthermore, interferon-gamma release assays for (1,3)-β-D-glucan were negative. Chest radiography revealed a mass in the right lower lung field (Figure 1A). Contrast-enhanced HRCT of the chest showed a 30 mm mass with a cavity in the posterolateral lower lobe of the right lung with a bronchial artery inside (Figure 1B,C). In addition, a new ground-glass opacity was observed in the left superior lung lobe, which was not present 3 weeks prior. Another nodule in the right middle lobe and mild enlargement of lymph nodes in the right neck, right hilar, and tracheal bifurcation were also observed.

**Figure 2:**
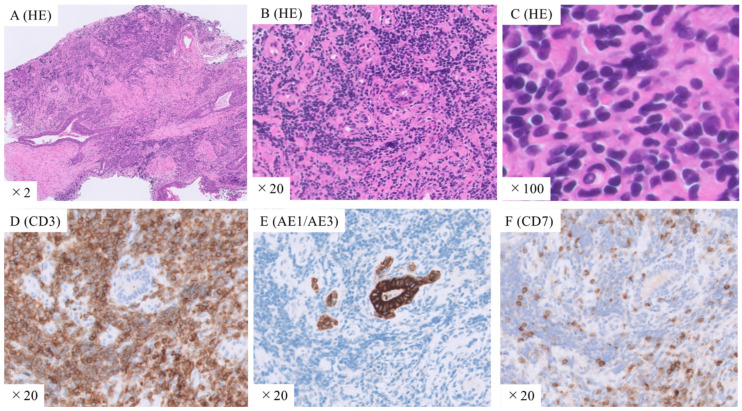
Histopathological images of lung tissues obtained using transbronchial lung cryobiopsy. (**A**–**C**) Hematoxylin and eosin staining shows diffuse proliferation of small-to-medium-sized lymphocytes with mild nuclear atypia. Immunostaining with (**D**) anti-CD3, (**E**) AE1/AE3, and (**F**) CD7 antibodies shows that the atypical lymphocytes are diffusely positive for CD3 and negative for AE1/AE3 and CD7. Original magnification: ×2 (**A**), ×20 (**B**,**D**–**F**), ×100 (**C**).

For definitive diagnosis, TBLC of the right lung lower lobe was performed. Additionally, endobronchial ultrasonography (EBUS) was performed to select the appropriate bronchi for tumor biopsy while avoiding vesicles (Figure 1D). TBLC was successfully performed with minimal bleeding and no major complications. Histological examination of the lung specimens revealed diffuse proliferation of small-to-medium-sized lymphocytes with mild nuclear atypia. The atypical lymphocytes were diffusely positive for CD3 and negative for AE1/AE3 and CD7 (Figure 2). In addition, the specimens were positive for CCR4. Therefore, the patient was diagnosed with ATL. He was referred to the hematology department for chemotherapy. Additional evaluation was also performed at the hematology department. Visual inspection, rather than machine-based measurement, revealed 3% of atypical lymphocytes in the peripheral blood. Positron emission tomography-CT showed uptake in lung lesions as well as in the lymph nodes of the right neck and mediastinum. No abnormal uptake was observed in other areas.

## 3. Discussion

The diagnosis of ATL is based on a combination of clinical symptoms, pathological findings, and confirmed HTLV-1 infection. Pathological diagnosis is essential, as abnormal lymphocytes are often positive for antibodies against CD3+, CD25+, and CCR4 [8]. A 78-year-old Japanese man was referred to our hospital because a definitive diagnosis could not be made using TBLB. TBLC with EBUS to avoid vesicles was performed without any major complications. The larger lung-tissue samples obtained using TBLC were helpful in making a definitive diagnosis of ATL.

To gather evidence and explore the usefulness of TBLC for diagnosing pulmonary lymphoma, we searched for published articles in the English and Japanese languages using Google and Google Scholar. In addition, the PubMed database and reference lists of selected articles were searched for relevant studies. Further, we used Ichushi-Web to search for Japanese papers. The selection criteria for our literature search encompassed the keywords “cryobiopsy” or “TBLC” in conjunction with “lymphoma” for both Japanese- and English-language reports. A total of 19 cases of pulmonary lymphoma were identified, including the present case (Table 1).

Of the 19 cases, 16 (84.2%) were diagnosed with B-cell lymphoma and three (15.8%) with T-cell lymphoma. The lymphoma subtypes were as follows: intravascular large B-cell lymphoma, five cases (26.3%); mucosa-associated lymphoid tissue (MALT), four cases (21.1%); diffuse large B-cell lymphoma, four cases (21.1%); Hodgkin’s disease, three cases (15.8%); T-cell lymphoma (not otherwise stated; one case, 5.3%); and peripheral T-cell lymphoma, one case (5.3%). The present case seems to be the first case of ATL diagnosed using TBLC. Prior and/or concurrent examination using TBLB was performed in three cases; however, a definitive diagnosis was not made without TBLC in any of these cases. Pathological examination of the small TBLB specimen suggested inflammatory lymphocytic infiltration. In three cases, EBUS was used in combination with TBLC to identify the location of the pulmonary lesion. In the present case, EBUS was used to avoid vascular biopsy. Of the nine cases with available data, seven (78%) underwent a biopsy of the middle or lower lobe. The number of specimens ranged from 2 to 13, with a minimum of two biopsied sites and no major complications in any case.

Pulmonary lymphomas are rare and account for 0.5–1% of all pulmonary malignancies. Most lymphomas are B-cell lymphomas, such as MALT and diffuse large B-cell lymphomas. MALT lymphomas and diffuse large B-cell lymphomas account for approximately 70% and 12–20% of cases, respectively, whereas pulmonary T-cell lymphomas account for approximately 10% of all pulmonary lymphomas [18]. In our review of 19 cases along with the present case, B-cell lymphoma accounted for 84.2% of the cases, whereas T-cell lymphoma accounted for 15.8%. The distribution of cases showed no significant deviation from that in previous reports. ATL is a globally rare T-cell lymphoma, although it is common in certain areas, including western Japan [2]. In a survey of lymphoma cases in Japan occurring over a 2-year period (2006 and 2007), ATL accounted for 910 cases (11.3%), whereas B-cell non-Hodgkin lymphoma accounted for 7164 cases (88.7%) [3]. The proportion of patients with ATL reflected the HTLV-1 positivity rate in each region, with a rate of 40.7% in the aforementioned areas. Because the prognosis for aggressive ATL is poor, with a median survival rate of 11 months and a 5-year survival rate of 14% [4], an early diagnosis and appropriate chemotherapy are essential.

Due to the relative rarity of lymphomas, management and diagnostic techniques are not well established. In addition, the difficulty in diagnosis is associated with damage to inflammatory lymphocyte infiltration due to cancer in small tissue specimens, such as those obtained using TBLB or CT-guided biopsy. Moreover, histological diagnosis is possible in only 30–50% of patients undergoing TBLB due to the absence of specific signs in several patients [19]. Although molecular analyses are useful for the diagnosis of lymphomas, this approach is difficult to apply to small samples. Thus, more than half of the patients are diagnosed through surgical intervention [5,20]. Surgical resection could be a treatment option for localized lymphomas, such as MALT lymphoma; however, ATL is a systemic disease, and chemotherapy is required for aggressive stages. Thus, the development of less invasive diagnostic procedures is crucial.

Specimens obtained using TBLC are five-times larger than those obtained using TBLB [21]. Thus, TBLC has recently been used for tissue biopsies in patients with diffuse lung disease and peripheral lung lesions. The 2022 Official ATS/ERS/JRS/ALAT Clinical Practice Guideline for Idiopathic Pulmonary Fibrosis recommends that TBLC should be considered as a viable alternative to SLB for histopathological diagnosis in patients with undetermined type of interstitial lung disease (ILD) in medical centers with experience in performing and interpreting TBLC. This recommendation is based on a systematic review of 40 studies evaluating TBLC in patients with an undetermined type of ILD where the diagnostic yield of TBLC was 79% [22]. In a prospective comparison involving 29 patients who underwent both TBLC and TBLB, TBLC exhibited a superior diagnostic yield compared to TBLB (69% vs. 38%) [21]. TBLC has also been applied in the diagnosis of lung cancer. In a study of 100 patients from Japan published in 2023, the diagnostic yields for TBLC, forceps biopsy, and brushing cytology were 86%, 81%, and 82%, respectively [23]. Lung cancer exhibits a high diagnostic rate even in cytology due to its cellular atypia. However, the size of its specimens suggests its utility for additional molecular biological investigations, such as programmed death-ligand 1 expression [24]. In terms of safety profiles, a systematic review in the statement for idiopathic pulmonary fibrosis shows that complications of TBLC included pneumothorax in 9% and any bleeding in 30% of cases. Severe bleeding, procedural mortality, exacerbations, respiratory infections, and persistent air leaks are rare [22]. The incidence of hemorrhage is not significantly different between TBLB and TBLC [25], and TBLC is relatively safer compared to SLB [10]. A systematic review and meta-analysis on the usefulness of TBLC for the diagnosis of diffuse lung disease, which included 297 and 150 patients who underwent TBLC and SLB, respectively, showed that TBLC decreased the median hospital stay (2.6 days versus 6.1 days) and mortality rate (0.3% versus 2.7%) [7]. In this review, 19 cases of pulmonary lymphoma were successfully diagnosed using TBLC without any major complications. However, in one case of T-cell lymphoma, the diagnosis could not be made using TBLC [26]. Nonetheless, the diagnostic utility and safety of TBLC for pulmonary lymphomas and ATL remain unclear. Therefore, prospective large-scale studies are required.

The present review demonstrated that 80% of patients underwent a biopsy of the middle or lower lobe. In practice, TBLC is considered particularly challenging for lesions in the upper lobe or for those with difficult branch selection due to maneuverability issues associated with inserting a hemostatic balloon. Recent studies from Europe on TBLC for diffuse lung diseases obtained lung samples from the lower lobes in over 80% of the cases [27,28]. Further, in a prospective study from Austria from 2018 to 2022 that was conducted after the establishment of standardized techniques evaluated the safety of TBLC in 75 individuals, and only 12.5% of the samples were taken from the upper lobes. Interestingly, TBLC was never performed to collect samples from the upper lobes in a cohort study conducted in Denmark between 2017 and 2020 that included 141 individuals [28]. However, a Japanese study on TBLC for the diagnosis of lung cancer between 2020 and 2021 showed that among 55 samples from 100 patients obtained from the upper lobes, the diagnosis was achieved in 44 (80.0%) [23]. Moreover, for the diagnosis of diffuse lung diseases, more than four samples were often obtained, and higher diagnostic concordance between TBLC and SLB samples was observed [27,28,29]. The diagnostic concordance between TBLC and SLB substantially increased when five TBLC samples were obtained compared to that with four TBLC samples (80% vs. 55%) [29]. A recent study from Japan showed that the diagnostic concordance between TBLC and SLB was 50%, 64%, and 75% for one, two, and three TBLC samples, respectively [30]. Furthermore, the 2022 Official ATS/ERS/JRS/ALAT Clinical Practice Guideline for Idiopathic Pulmonary Fibrosis, which evaluated 40 studies on TBLC, states that the diagnostic yield is 85% when three or more samples are collected, but it decreases to ≤77% when fewer samples are collected [22]. In contrast, in a recent study of TBLC for diagnosing lung cancer, even an average of 1.1 samples showed a high diagnostic rate. The diagnostic yield by TBLC was 86%. In the present review of 19 cases of pulmonary lymphoma, Bianchi et al. and Poletti et al. [9,13] collected a larger number of samples more aggressively using rigid bronchoscopy (Table 1), whereas other researchers collected two or three samples. Therefore, further investigations are required to determine the biopsy location and optimal number of samples for the diagnosis of pulmonary lymphoma.

EBUS can be used for visualizing the interior of a lesion and determining the biopsy site [31]. Furthermore, it can be used during TBLC for site assessment before cryobiopsy, thereby avoiding the vasculature and reducing the risk of complications. In a multicenter prospective study involving 87 patients with diffuse lung disease, 49 patients underwent TBLC with EBUS. These patients more frequently experienced no or mild bronchial bleeding (91.8%) compared to those who did not undergo EBUS (65.8%) (*p* < 0.01). Additionally, the procedure duration was shorter in the EBUS-assisted group (median, 31 min versus 37 min; *p* < 0.01) [32]. In our review, EBUS was used in combination with TBLC in three cases to identify the location of pulmonary lesions. In the present case, the bronchus passed through the tumor without narrowing. The ultrasound probe inside the tumor showed that the bronchial vessels maintained a regular circular shape and were pulsating. In such cases, it is difficult to collect specimens using TBLB, and TBLC, which can collect large specimens by freezing, is beneficial. In the present case, EBUS was also useful for avoiding vascular biopsy. Accordingly, the findings in the present case suggest that the combination of TBLC and EBUS may be useful for obtaining samples of higher diagnostic quality and size for the diagnosis of pulmonary lymphoma and for achieving hemostasis.

## 4. Conclusions and Future Directions

We presented the first case of ATL diagnosed using TBLC. To compile evidence, we conducted a literature review on the application of TBLC and identified 18 cases of pulmonary lymphomas. Among the 19 cases, including the present case, 16 patients were diagnosed with B-cell lymphoma (84.2%). Three cases, including the present ATL case, were T-cell lymphomas. Pulmonary lymphoma was successfully diagnosed using TBLC in 19 patients, of whom 80% underwent biopsy in the middle or lower lobe, and more than two samples were obtained without any major complications. EBUS was used in combination with TBLC in three cases to identify the location of the pulmonary lesions. Although large-scale prospective studies are required to establish precise guidelines for diagnosing pulmonary lymphomas using TBLC, our case report and review of cases in Japan and internationally contributes to a deeper understanding of the approach to diagnosing rare diseases.

## Figures and Tables

**Table 1:** Diagnoses of pulmonary lymphoma through transbronchial lung cryobiopsy reported in the relevant English and Japanese literature, including the present case.

Authors	Publication	Pathology of Lymphoma	TBLB	EBUS	Site	N	C
Poletti V. et al. [9]	2015	Intravascular large B-cell	−	−	RLL	4	−
Poletti V. et al. [9]	2015	Intravascular large B-cell	−	−	RLL	2	−
Schiavo D. et al. [10]	2016	Diffuse large B-cell	+	−	LUL	3	−
Yap E. et al. [11]	2017	Diffuse large B-cell	−	+	RML	2	−
Sato M. et al. [12]	2020	T-cell	+	+	RLL	2	−
Bianchi R. et al. [13]	2020	MALT	NA	NA	NA	5	−
Bianchi R. et al. [13]	2020	Peripheral T-cell	NA	NA	NA	5	−
Bianchi R. et al. [13]	2020	MALT	NA	NA	NA	4	−
Bianchi R. et al. [13]	2020	Hodgkin	NA	NA	NA	7	−
Bianchi R. et al. [13]	2020	MALT	NA	NA	NA	2	−
Bianchi R. et al. [13]	2020	MALT	NA	NA	NA	5	−
Bianchi R. et al. [13]	2020	Hodgkin	NA	NA	NA	4	−
Bianchi R. et al. [13]	2020	Diffuse large B-cell	NA	NA	NA	13	Bleeding (moderate)
Bianchi R. et al. [13]	2020	Lymphoplasmocytic	NA	NA	NA	9	−
Zhu D. et al. [14]	2021	Intravascular large B-cell	−	−	NA	NA	−
Tan CH. et al. [15]	2022	Hodgkin	−	−	LUL	2	−
Michimata H. et al. [16]	2022	Intravascular large B-cell	−	−	RLL	NA	−
Uchimura K. et al. [17]	2022	Intravascular large B-cell	−	−	RLL	NA	−
Tanaka Y. et al. (Present case)	2023	ATL	+	+	RLL	2	−

Abbreviations: ATL, adult T-cell lymphoma; C, complication; EBUS, endobronchial ultrasound; LUL, left upper lobe; MALT, mucosa-associated lymphoid tissue; RML, right middle lobe; NA, not applicable; N, number of samples; TBLB, prior or concurrent examination using transbronchial lung biopsy; Site, biopsy site.

## Data Availability

Data will be provided upon reasonable request.
